# Perception of quality of life by children and adolescents with cleft lip/palate after orthodontic and surgical treatment: gender and age analysis

**DOI:** 10.1186/s40510-021-00354-8

**Published:** 2021-04-01

**Authors:** Ana Ruiz-Guillén, Carlos Suso-Ribera, Martín Romero-Maroto, Carmen Gallardo, Cecilia Peñacoba

**Affiliations:** 1 Department of Nursing and Dentistry, Rey Juan Carlos University, Madrid, Spain; 2 Pediatric Dentist, Private Practice, Madrid, Spain; 3 Department of Basic and Clinical Psychology and Psychobiology, Jaume I University, Castellón, Spain; 4 Orthodontic Department, Rey Juan Carlos University, Madrid, Spain; 5 Department of Medical Specialties and Public Health, Rey Juan Carlos University, Madrid, Spain; 6 Deparment of Psychology, Rey Juan Carlos University, Madrid, Spain

**Keywords:** Quality of life, Cleft lip and/or palate, Children, Adolescents

## Abstract

**Background:**

The quality of life (QoL) of children and adolescents with cleft lip/palate (CL/P) has been shown to be a predictor of good psychosocial functioning in this population group. This study aimed to measure QoL, from the patient´s perception of change produced by the different surgical and orthodontic treatments carried out since early childhood, and if gender and age are modulating the outcome variables results.

**Materials and methods:**

A cross-sectional research study was carried out. The study included 60 patients with cleft lip, cleft palate, or cleft lip/palate, aged between 8 and 18, who were in orthodontic treatment and had undergone at least one surgery. They were asked to complete the Quality-of-Life Adolescent Cleft Questionnaire (QoLAdoCleft), which allows the assessment of the QoL through self-perception of improvement after surgical and orthodontic interventions. In particular, this questionnaire (administered only once), allows the evaluation of self-perception of QoL at the present time and before orthodontic and surgical treatment. This double assessment was carried out for the domains of physical, psychological, and social health. The results were analysed by looking at the interaction of gender and age.

**Results:**

Statistically significant differences were found in the perception of the current QoL in comparison to the retrospective perception in all the dimensions considered. The perception of QoL improved in all cases. The results also showed a moderation of gender in the relation between perception of previous behaviour and social function and actual behaviour and social function.

**Conclusion:**

The results indicated that patients perceived their quality of life had improved as a result of the treatments received, with the highest effect sizes found in the physical health domain. Specifically, the improvement in QoL in behaviour and social function tended to be influenced to a greater extent by perception of previous QoL. In this sense, personalized preventative measures from holistic and biopsychosocial approaches are necessary.

## Introduction

Cleft lip/palate (CL/P) is a congenital anomaly that leads to problems with facial appearance and function (e.g. chewing, swallowing, hearing, and speech) [1–4]. Facial malformations and the conditions of their treatment, which are considered as stress factors, can have potentially significant consequences for the lives of these patients [5]. Physical attractiveness is an important psychological variable in the field of appearance, which influences child development [6]. For this reason, children and adolescents with CL/P can be a particularly vulnerable group [2].

The study of oral health-related quality of life (OHRQoL) is the most used in the field of CL/P, constituting an indicator of success of medical interventions [3, 4]. Information on OHRQoL helps rehabilitation specialists understand the burden of disease from the patient’s perspective and thus improve treatment recommendations [7].

Treatment of CL/P affects many domains related to a patient’s QoL, including appearance, speech, self-image, social integration, and physical and psychological functioning [8].

The treatment of CL/P patients leads to an improvement in their emotional states related to body image, and it also increases self-esteem, self-confidence, and social competence, all of which have positive effects on increasing QoL [1, 6, 9]. For this reason, the treatment is focused on achieving excellent functional, but also aesthetic, results that improve the QoL of patients with CL/P [8].

Treatment protocols include numerous surgical and orthodontic treatments from birth until adulthood [10]. The first phase of the treatment lies in placing presurgical infant orthopedics such as Nasoalveolar Molding or Latham-Millard [11, 12], or lip adhesion through the use of adhesive strips [13], these treatments improve surgical outcomes as they facilitate tension-free closure of the lip, improved nasal symmetry, and allow potential soft tissue surgical unification of the alveolar segments through a gingivoperiosteoplasty at approximately 3 months of age [12]. In cases of a cleft palate, at about 9–12 months of age, palatoplasty is performed [14]. Depending on the degree of malocclusion, these patients need orthodontic palatal expansion and/or maxillary protraction [15], combined with bone grafting for the closure of the oronasal communication, in which case several grafts may be necessary [13, 16]. Treatment continues with a fixed orthodontics appliance (brackets) [17]; in some cases, it is necessary to perform a surgical treatment the Le Fort 1 and sagittal osteotomy of the mandible [13].

Taking into account studies that have demonstrated the impact that treatment has on the individual [1, 2, 5, 18, 19], a multidisciplinary approach is necessary, as patients’ mental state, their expectations regarding the treatment, and the effects of the treatment on their well-being need to be considered [5]. In this context, there are studies that assess the QoL of children and adolescents with CL/P using specific instruments for this pathology, such as the Autoquestionnaire Qualitée de Vie Enfant Image [20], CLEFT-Q [21]^,^ or THAICLEFT QoL Questionnaire [22]; however, these instruments are usually used to measure this variable at a single moment in time, with ‘pre-post’ designs being less common. Although studies that measure this variable before and after treatment (at two time points) can also be found, such as the study carried out by Beluci and Genaro (2016) [1] and Antoun et al. (2015) [23], the instruments used are not specific for patients with a cleft.

An interesting approach in this sense is that proposed by Piombino et al. [24] through their questionnaire Quality of Life Adolescent Cleft Questionnaire (QoLAdoCleft). This questionnaire allows an assessment (at a single point in time) of the current QoL of the patient, together with a retrospective perception of their pre-treatment QoL, therefore providing information on the patient's perception of improved QoL treatment.

Thus, the aim of the current cross-sectional study has been to assess both the perception of current and retrospective QoL before treatment among children and adolescents with CL/P, taking into account functional, aesthetic, psychological, and social dimensions. Additionally, the study aimed to examine whether gender and age influence the results.

## Methods

### Sample characteristics

We recruited 60 children and adolescents with CL/P (*M*_age_ = 12.80 years; SD = 2.79; age range = 8–18 years; 27 males and 33 females) who were attending orthodontic treatment in two private health centres in Madrid. Specifically, participation by age was as follows: 8 years (*n* = 2; 3.3%), 9 years (*n* = 4; 6.7%), 10 years (*n* = 11; 18.3%), 11 years (*n* = 6; 10%), 12 years (*n* = 9; 15%), 13 years (*n* = 5; 8.3%), 14 years (*n* = 3; 5%), 15 and 16 years (*n* = 7; 11.7% respectively), 17 years (*n* = 5; 8.3%), and 18 years (*n* = 1; 1.7%).

Approximately 17% (*n* = 10) had a diagnosis of isolated cleft lip, 23.3% (*n* = 14) had isolated cleft palate, and 60% (*n* = 36) had CL/P. Ninety per cent (*n* = 54) had undergone more than one surgery. The mean age at which they underwent the first surgery was 5.83 months (SD = 3.97), and 36.07 months (SD = 37.69) for those who underwent a second surgery.

All patients were undergoing orthodontic treatment. According to the treatment protocol for patients with a cleft, thirty-three patients in the sample (55%), who were in mixed dentition, were being treated with a Hyrax expander, twenty of these also needed a face mask for early treatment of Class III malocclusion. Twenty-seven patients (45%) were in treatment with fixed braces. If the malocclusion was not solved with orthodontic treatment, these cases would have to be prepared for orthognathic surgery. The treatment duration varied among patients, depending on the severity of the malocclusion.

Since most were in the middle of orthodontic and/or surgical treatment, post-occlusal outcome was not included as a variable in this study.

### Eligibility criteria

The inclusion criteria for the sample were to be between 8–18 years old, in orthodontic treatment and to have undergone previous surgery to improve their appearance and/or functionality. Patients with a syndrome-associated cleft that could interfere with their intellectual or cognitive ability were excluded.

The sample age was chosen so that the patients were already in mixed dentition, and also had an appropriate level of understanding to answer the questionnaire. Moreover, at the age of 8, social relations become more important and can influence QoL [23]. Although the questionnaire has been previously validated (by our research team) for patients aged 8–18, children were assisted by a researcher who made sure they understood the answer format, without influencing the children answers.

During the sampling period, 75 patients met the inclusion criteria; however, sixty (80%) completed the self-report questionnaire in full. The reason for non-completion was that parents, or the patients themselves, did not consider that they had any psychological adjustment problem.

### Data collection

Data was obtained through self-reported questionnaires that patients filled out during their orthodontic check-ups. The study was conducted from 2016 to 2018.

Participants self-reported their gender and age at the time the questionnaires were collected. The convenience sample consisted of 60 consecutive patients, from two geographical areas of two health centres, specialized in the treatment of cleft.

The study followed the guidelines of the research ethics committee of the Rey Juan Carlos University. No. 110720166716 , and informed consent was obtained from the patients’ parents.

### Measures

#### Quality of life

The Spanish adaptation of the Quality-of-Life Adolescents Cleft Questionnaire (QoLAdoCleft) was used [24]. Specifically, the QoLAdoCleft allows for both the assessment of current QoL and the retrospective perception of it before treatment in three domains: physical, psychological, and social health. In terms of physical health, the dimensions of physical function were measured (4 items: chewing, regurgitation, associated ear, and breathing pathologies; Cronbach’s alpha = .75), communication (2 items: pronunciation, understanding by others; Cronbach’s alpha = .80), and pain (1 item: existence of triggers points). For psychological health, the domains of self-concept (2 items: feeling less valid or different from others, Cronbach’s alpha = .87) and behaviour (1 item: tendency to isolate) were measured. For social health, the social function domain was measured (1 item: influence of physical appearance on social activities). These items were intended to evaluate the patient’s condition before and after orthodontic and surgical treatment, although the questionnaire was administered only after physical and/or functional improvement of the orthodontic and surgical treatment in all cases.

Due to the wide range of ages covered in this study, the level of improvement differed between patients because they were at different stages of treatment, and the information obtained was based on subjective assessment of the health status of the individual.

### Data analysis

First, perception of improvement of QoL due to treatment was analysed using a Student´s *t-*test for related samples. Second, moderation analyses were conducted with model 1 from the PROCESS Macro version 3.4 [25]. Age and gender were used as moderators, QoL dimensions before treatment as independent variables, and QoL dimensions after treatment as the outcomes. Twelve models were tested (six for gender and six for age as moderators), two for each QoL dimension. Statistical significance was set at an alpha level of 0.05. In the PROCESS Macro, for the continuous variable (i.e. age), the recommended values in conditional tables and graphical representations are the 16th, 50th, and 84th percentiles. Thus, these cut-offs were used to calculate conditional effects (i.e. effects of an independent variable on an outcome for different values of a moderator) in the case of a significant moderation effect.

## Results

### Perception of improvement of QoL due to treatment

As can be seen in Table 1, all QoL dimensions assessed showed statistically significant differences. QoL was perceived to have improved after treatment in all cases. In terms of the effect size, the highest improvements were observed for the physical function and communication dimensions, whereas pain showed small effect sizes. Improvements in psychological and social health showed moderate effect sizes.

**Table 1:** Perception of improvement in the QoL dimensions after orthodontic and surgical treatment

	Before Mean (SD)	After Mean (SD)	*t*	*p*	*d*-Cohen
**Physical health**					
Physical function	6.63 (4.01)	3.20 (2.44)	8.083	< .000	1.03
Communication	3.85 (2.50)	2.03 (1.86)	6.850	< .000	.82
Pain	.61 (.85)	.34 (.71)	2.519	.015	.34
**Psychological health**					
Self-concept	3.02 (2.60)	2.03 (2.08)	4.448	< .000	.42
Behaviour	1.16 (1.31)	.70 (.99)	3.746	< .000	.40
**Social health**					
Social function	1.13 (1.15)	.60 (.90)	4.361	< .000	.51

### Regression and moderation analyses with gender as moderator

The results of the regression analyses, including the analysis of moderation effects (gender), are presented in Table 2. The prediction of QoL dimensions after treatment from their respective QoL dimensions before treatment, gender, and their interaction demonstrated significant direct contributions of each dimension before treatment on their respective dimension after treatment. However, we found no direct effect of gender on QoL dimensions after treatment. The results showed a moderation of gender in the relation between behaviour before treatment and behaviour after treatment (beta = − 0.31, *t* = − 2.28, *p* = .02, 95% confidence interval [CI]: − 0.59, − 0.04) and between social function before treatment and social function after treatment (beta = − 0.33, *t* = − 2.09, *p* = .04, 95% CI: − 0.64, − 0.01).

**Table 2:** Prediction of quality of life after orthodontic and surgical treatment from quality of life before treatment, gender, and their interaction

	*R*^2^	*F*	*p*	Beta	*t*	*p*	95% CI
DV = Physical function	0.36	10.36	< .000				
Physical function (before)				0.38	4.46	< .000	0.21, 0.56
Gender				− 0.75	− 1.44	.15	− 1.80, 0.29
Interaction				− 0.06	− 0.47	.63	− 0.33, 0.2
DV = Communication	0.36	10.54	< .000				
Communication (before)				0.40	3.67	< .000	0.18, 0.62
Gender				− 0.26	− 0.66	.51	− 1.07, 0.54
Interaction				0.09	0.61	.54	− 0.22, 0.41
DV = Pain	0.22	5.19	.003				
Pain (before)				0.46	3.24	.002	0.18, 0.75
Gender				− 0.15	− 0.87	.39	− 0.50, 0.19
Interaction				− 0.13	− 0.66	.51	− 0.55, 0.27
DV = Self-concept	0.57	25.04	< .000				
Self-concept (before)				0.62	7.38	< .000	0.45, 0.79
Gender				− 0.10	− 0.28	.78	− 0.84, 0.64
Interaction				− 0.08	− 0.52	.60	− 0.39, 0.23
DV = Behaviour	0.54	21.66	< .000				
Behaviour (before)				0.68	7.06	< .000	0.49, 0.88
Gender				− 0.31	− 1.74	.08	− 0.68, 0.05
Interaction				− 0.31	− 2.28	.02	− 0.59, − 0.04
DV = Social function	0.44	14.59	< .000				
Social function (before)				0.63	5.89	< .000	0.41, 0.84
Gender				− 0.32	− 1.79	.08	− 0.69, 0.04
Interaction				− 0.33	− 2.09	.04	− 0.64, − 0.01

**Fig. 1:**
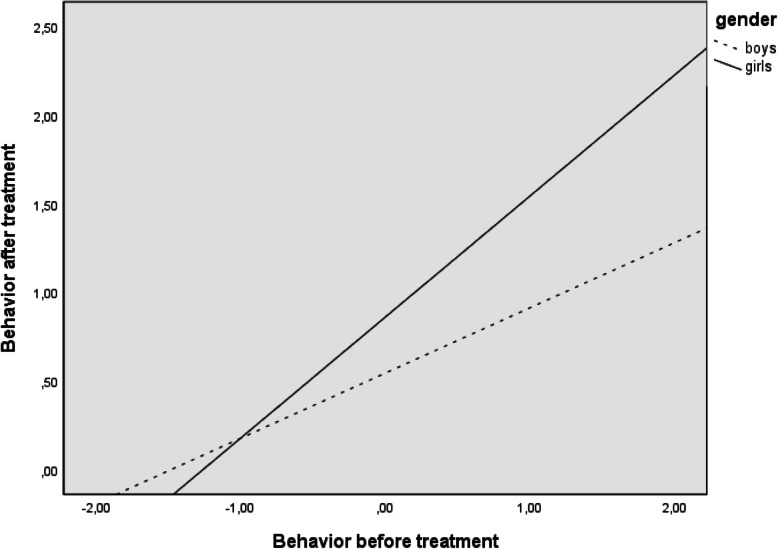
Relationship between behaviour before and after treatment depends on gender

**Fig. 2:**
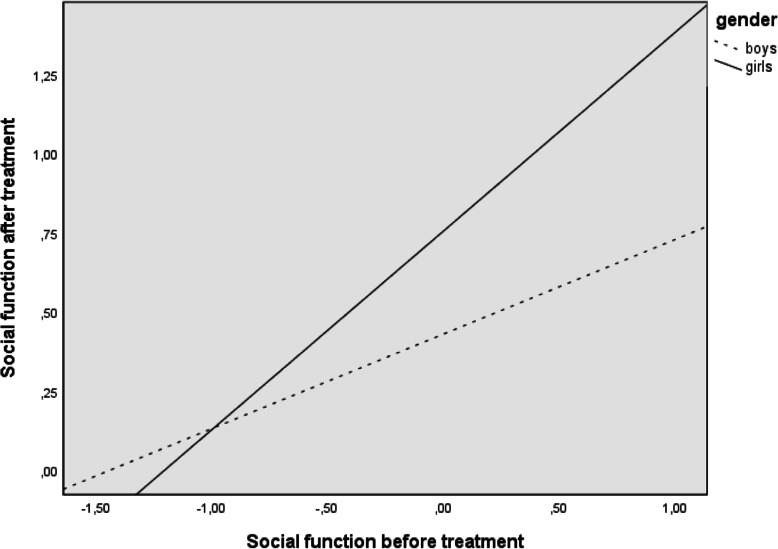
Relationship between social function before and after treatment depends on gender

As noted earlier, post hoc analyses were planned to examine significant moderations in more depth. Table 3 and Fig. 1 show the results for the moderation of gender in the relation between behaviour before treatment and behaviour after treatment, whereas Table 4 and Fig. 2 give those for the moderation of gender in the relation between social function before treatment and social function after treatment. As noted, behaviour and social function before treatment had a higher predictive capacity for behaviour and social function after treatment for girls than boys.

**Table 3:** Conditional effects of behaviour before orthodontic and surgical treatment on behaviour after treatment depend on gender

Gender	Beta (social function before)	*t*	*p*	95% CI
Girls	0.683	7.066	< .000	0.49, 0.88
Boys	0.368	3.723	< .000	0.17, 0.57

**Table 4:** Conditional effects of social function before orthodontic and surgical treatment on social function after treatment depend on gender

Gender	Beta (behaviour before)	*t*	*p*	95% CI
Girls	0.627	5.891	< .000	0.41, 0.84
Boys	0.299	2.576	.012	0.06, 0.53

### Regression and moderation analyses with age as moderator

The prediction of QoL dimensions after treatment from their respective QoL dimensions before treatment, age, and their interaction revealed significant direct contributions from each of the dimensions before treatment on their respective dimension after treatment. No moderation effect of age on QoL dimensions after treatment was observed. The results showed a direct effect of age on social function after treatment (beta = − 0.10, *t* = − 3.23, *p* = .002, 95% CI: − 0.17, − 0.04), indicating that older patients reported better social function after treatment.

## Discussion

The main objective of this study was to analyse whether QoL increases in children and adolescents with CL/P after their aesthetics and functionality have been treated with orthodontics and surgery. The results showed that patients perceive that their current QoL has increased after orthodontic and surgical treatment, in relation to all the evaluated domains (physical, psychological, and social health). The largest effect sizes were found in physical health, specifically in physical function and communication.

Although these results are generally consistent with those found in the literature, it is difficult to make specific comparisons by dimension, given the heterogeneity of measures used for the evaluation of QoL with regards to the instruments being not specific to children and adolescents with this condition.

Among the general QoL assessment instruments applied to CL/P, the Oral Health Impact Profile-14 (OHIP-14) should be highlighted owing to its high frequency of use. Thus, with this instrument, Beluci and Genaro [1] found QoL increases after treatment in the physical, psychological, and environmental domains (in line with our results), but reported no significant differences in social relationships before and after treatment. However, Antoun et al. [23], also using OHIP-14, reported a small change in OHRQoL pre-post treatment in a CL/P sample (in comparison with outcomes of surgical patients without a cleft). This small change is argued on the basis that cleft patients do not experience a drastic transformation of their appearance compared to other surgical patients. Broder et al. [2], using the Child Oral Health Impact Profile, found that surgical youth with a cleft experienced the greatest increase in OHRQoL in comparison with non-surgical youth with CL/P.

Other results using general QoL instruments (e.g. Michigan Oral Health-Related Quality of Life Scale) after treatment, in samples between 12 and 23 years of age, indicated adequate levels of QoL after treatment, particularly in the domain of oral functionality, consistent with our results [5].

As noted, other studies have used specific instruments to measure QoL in patients with CL/P. These include the questionnaire designed by Piombino et al. [24], whose adaptation we used in the present study. To our knowledge, this instrument is the only one that, administered only once, after the intervention, allows an assessment of the patient’s perception, before and after treatment, of different domains of QoL. The results, in an age sample of 16 to 23 year olds, showed improvements in the physical health domain and in psychological health (specifically the behaviour dimension), although they did not make a differential analysis of the effect sizes on the perception of improvement in the different domains [24].

The differential study of the self-perception of QoL improvement after treatment is a novel aspect of this research. Our results show that this self-perception of improvement (assessed at a single moment in time through both a current and a retrospective perception) coincides, as a whole, with the results of improved QoL observed in studies with ‘pre-post’ treatment designs [1].

In this context, effective treatment is important, particularly surgery, as its effect is a possible improvement on self-perception [19]. Few studies have placed value on the improvement in the psychological and social health areas in relation to the physical area after treatment in children and adolescents. Moreover, the few results in this regard are not conclusive. Albers et al. [19], in a population with CL/P (aged 12 to 63 years), observed a reduction in dissatisfaction with facial appearance after nose surgery, but found no significant change in self-concept in the short term. They concluded that, after years of psychological adaptation to the malformation, relatively small changes from functional and cosmetic surgery can result in a significant reduction in distress and increased psychological well-being. However, other studies have predicted improvements in self-concept after treatment [18, 26]. Self-concept has been conceptualized as a central element in most studies, looking at improving OHRQoL in children with CL/P after treatment [27].

Our results indicate significant ‘pre-post’ treatment improvements in all of the assessed QoL areas, from the young patients’ retrospective perception. Consistent with previous studies, the highest effect sizes were observed in physical health, particularly in physical function and communication, as they involve the direct results of the intervention itself. The psychological (self-concept and behaviour) and social health domains showed moderate effect sizes. In this context, and given the absence of studies carried out from this pre-post retrospective design in children and adolescents, future research should be directed at the mechanisms of improvement in the psychological and social areas after treatment.

Finally, another aspect of interest in this study, which has been absent in previous ones, is the possible interaction of gender and age in pre-post treatment improvements across the different domains of QoL. Regarding the interaction of gender in these domains, no direct effects on QoL were found after treatment improvement; however, a gender interaction was observed in the relation between the domains before and after treatment, particularly in behaviour and social function. Specifically, perception of post-treatment QoL was more influenced by perception of previous QoL in girls than in boys. This finding has important practical repercussions, as the effects of treatment on QoL will depend on perception of previous QoL to a greater extent in girls than in boys. Thus, differential and personalized preventative treatment actions must be applied in different domains. To our knowledge, no previous studies have specifically addressed this issue. However, although unrelated to treatment, different authors [2, 3, 10, 27] have reported greater perception of aesthetic and behavioural problems, dissatisfaction with their image, low self-concept, and emotional instability in women than in men with CL/P, and particularly, in the young and adolescent population. The differences with regards to social function are controversial, with studies indicating better [28] and worse scores [10] for women than for men in this domain.

Regarding age, no interaction effects were observed in the relation between QoL perceptions before and after treatment. We only found that social functioning after treatment improved with the age of the children and adolescents. However, while some studies have pointed to a better psychosocial adjustment in older adolescents [3], others have found worse psychosocial adjustment at a later age [23, 29]. These results could be explained by the disparity in the instruments and age ranges considered; in any case, none of the preceding studies have evaluated the influence of age on the relationship of QoL before and after treatment.

The present study has a number of limitations that need to be considered. The associations must be interpreted according to the observational nature of the study design, which does not allow inferences of causality. Although the advantage of the instrument used for the measurement of QoL, compared with the rest of the existing ones, lies in its assessment of variables before and after the treatment, it did so retrospectively, being administered at a single moment in time in our study. It is important to note that this assessment is not equivalent to the administration of the same instrument at two different times (before and after the intervention). In particular, the retrospective measure (especially the pre-treatment moment) could lead to an overestimation bias of the current QoL compared with the previous one.

Furthermore, the instrument does not allow to assess which treatment (surgical and/or orthodontic) produces the perception of improvement. The age range considered was wide, although relatively narrow compared with other studies [18, 19]. However, with regards to this limitation, our results indicated the absence of significant relations (except in the case of social function) between age and QoL. Finally, we should add the non-consideration of the type of cleft, and therefore the type of malocclusion, as an analysis variable (owing to the size of the sample), which made it impossible to evaluate the results according to the severity of the malformation. Nonetheless, the dimensions of QoL contemplated are common to all types of clefts. Thus, we encourage researchers to replicate these findings using different populations with CL/P and including other important variables such as clinical orthodontic outcomes.

## Conclusion

The present findings have important clinical implications. The study found relative improvements in the QoL of children and adolescents with CL/P after receiving treatment compared with their perception before treatment. The physical function domain showed the best results, which in turn may influence the positive effects on psychological and social health. We also found that improvements in QoL, in terms of behaviour and social function after treatment, could be influenced more by their respective levels (pre-treatment) in the case of girls. In this sense, personalized preventative measures from holistic and biopsychosocial approaches are necessary.

## Data Availability

The datasets used and/or analysed during the current study are available from the corresponding author on reasonable request.

## Abbreviations

QoL: Quality of life
CL/P: Cleft lip/palate
OHRoL: Oral health-related quality of life
QoLAdoCleft: Quality of life adolescents questionnaire
OHIP-14: Oral health impact profile-14
